# Institutionalizing Health Literacy as a Governance Mechanism in Low- and Middle-Income Countries: A Transformative Roadmap from the MENA Region

**DOI:** 10.34172/hpp.45753

**Published:** 2026-06-06

**Authors:** Parisa Hajibadali, Hamid Allahverdipour

**Affiliations:** ^1^Student Research Committee, Tabriz University of Medical Sciences, Tabriz, Iran.; ^2^Department of Health Education and Promotion, Faculty of Health, Tabriz University of Medical Sciences, Tabriz, Iran; ^3^Research Center of Psychiatry and Behavioral Sciences,Tabriz University of Medical Sciences, Tabriz, Iran

**Keywords:** Capacity building, Empowerment, Health in All Policies (HiAP), Health governance, Health literacy, Low- and middle-income countries (LMICs)

## Abstract

Health literacy (HL) is increasingly recognized not merely as an individual asset, but as a structural determinant of health equity and a critical governance instrument for sustainable health systems. While frameworks for "health literate organizations" are well-established in high-income contexts, low- and middle-income countries (LMICs) face similar systemic barriers—including centralized governance, fragile social trust, and resource constraints—that require distinct implementation roadmaps.In this perspective we argue that for health systems in the Middle East and North Africa (MENA) region to succeed, they must pivot from a didactic, education-centric paradigm toward an empowerment-oriented governance model. Accordingly, we articulate a multidimensional framework structured around five interdependent pillars: 1. structural reform and high-level advocacy, 2. mobilizing social capital and trust, 3. human resource empowerment, 4. primary health care transformation, and 5. sustainable financing. Drawing on global evidence—including WHO frameworks, the Ottawa Charter, and Health in All Policies—this paper proposes a multidimensional roadmap that institutionalizes HL as a core accountability mechanism within reginal health governance in the MENA region. This model offering a politically astute and transferable framework for LMICs seeking to embed health equity within the architecture of the state.

## Introduction

 Health literacy (HL) is no longer a peripheral educational goal but a core governance priority that underpins social justice and sustainable health systems.^1^ In its modern conceptualization, HL extends beyond the functional ability to read and understand health information; it encompasses the critical capacity of citizens to access, appraise, and apply health knowledge to make informed decisions and act effectively within their social and environmental contexts and active engagement in health-promoting actions.^2^ It is intrinsically linked to empowerment. Increasing HL is considered a critical strategy to empower individuals to gain more control over their health and its determinants.^2,3^ HL has become a prominent issue on the agenda of organizations like the World Health Organization (WHO), which positions it as a key pillar for achieving sustainable development and health equity.^1^

 While organizations like the World Health Organization (WHO) position HL as a key pillar for achieving the Sustainable Development Goals (SDGs), implementation science remains heavily skewed toward the Global North.^3^ Existing frameworks, such as the seminal work by Sørensen et al. (2012), effectively define what a health literate system looks like, but often lack specific guidance on how centralized health systems in transitional economies can operationalize these concepts amidst structural constraints.^4^

 In the Middle East and North Africa (MENA) region and similar LMIC contexts, persistent inequities—driven by sociodemographic disparities and limited institutional trust —underscore the urgent need for a governance-based HL transformation. It is important to acknowledge the heterogeneity within the MENA region, which encompasses diverse political systems, a wide economic spectrum ranging from high-income Gulf states to resource-limited economies, and varying degrees of stability including conflict-affected zones.^5,6^ In Iran, for instance, national surveys and systematic reviews consistently demonstrate limited health literacy, with more than 50% of adults exhibiting inadequate or marginal levels, a figure strongly associated with structural inequities rather than mere individual knowledge deficits. ^7,8^ This pattern is reinforced by recent multi-country evidence; a cross-sectional study of 4,909 participants across six Middle Eastern nations (including Egypt, Jordan, and Lebanon) found that a striking 75.1% of the population demonstrated problematic health literacy, particularly in emergency contexts.^9^ Furthermore, in Kuwait, nearly 44.5% of chronic disease patients exhibit inadequate HL, directly impeding clinical outcomes. ^10^ These figures highlight a systemic failure, wherein fragmented governance and a lack of institutional accountability systematically produce and reproduce low health literacy, transforming it from an individual risk factor into a population-wide marker of structural neglect.^11^

 Consequently, the roadmap proposed here, addresses not just a national concern, but a regional governance crisis. This perspective argues that health governances across the MENA region, must shift from a “deficit model” (fixing patients) to a “strengths-based governance model” (fixing systems). We propose a Systemic Roadmap for Capacity Building, framing HL as a metric of institutional accountability rather than patient compliance.

###  Conceptual Framework: Moving from Education to Empowerment 

 Unlike traditional Western models that often focus on organizational attributes (e.g., signage, hospital navigation), our proposed framework conceptualizes Transformative Health Literacy as a political and societal change process. It rests on two complementary dimensions tailored for developing health systems:

1. System and professional Empowerment: Enhancing the competence, motivation, communication skills, and accountability of the health workforce.
2. Community and Civic Empowerment: Enabling citizens and communities to engage in advocacy, participate in health governance, and demand equitable access and quality care.

**Figure 1:**
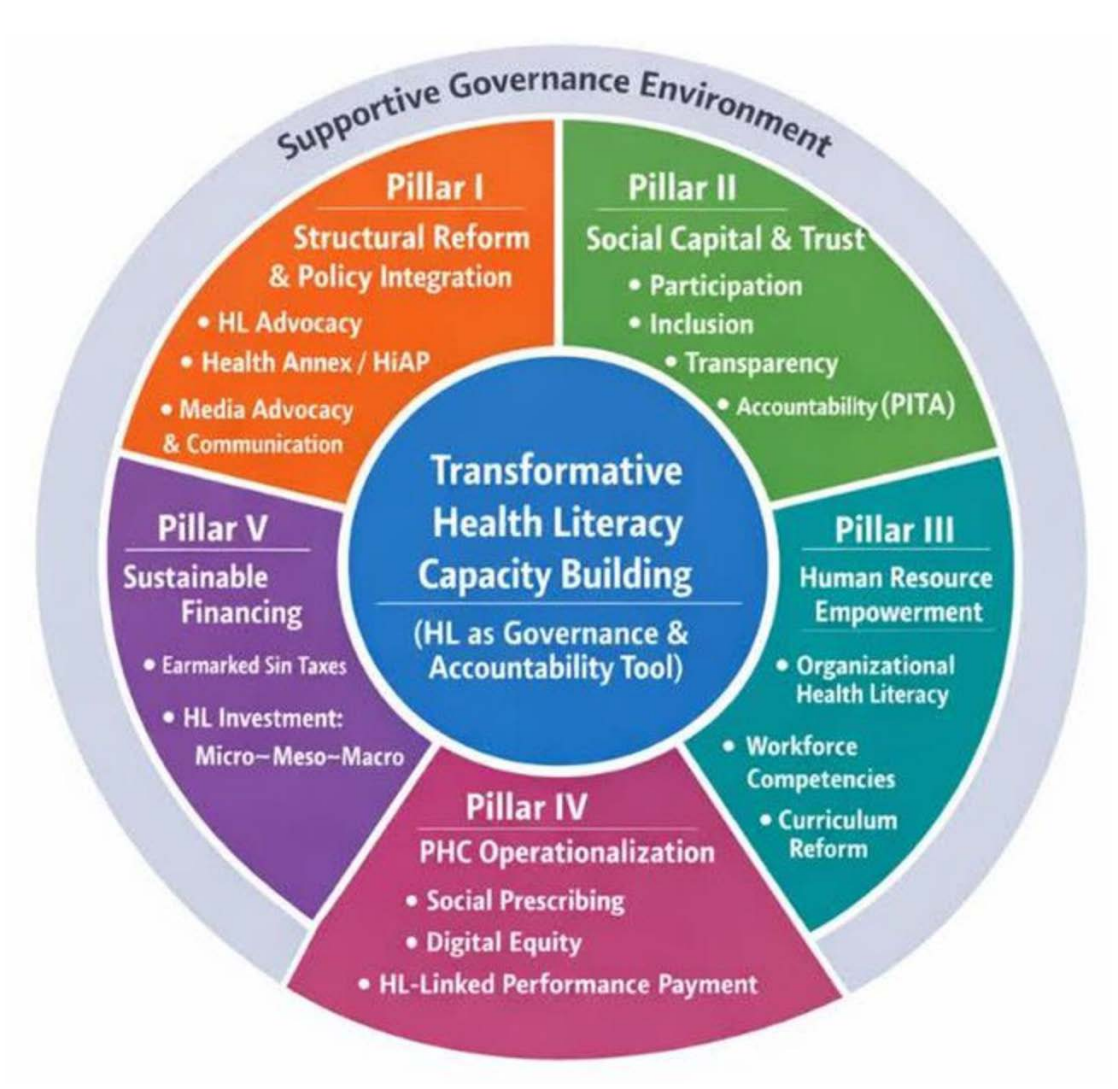
Governance-based systemic roadmap for transformative health literacy capacity building in LMICs. Health literacy is positioned as a core accountability mechanism, supported by five interdependent pillars operating across policy, organizational, community, and financing domains

As illustrated in in Figure 1, capacity building for HL sits at the center, supported by five interdependent pillars. This model operationalizes the Shanghai Declaration’s mandate to “promote health literacy as a political choice”.^12^

## Pillar I: Structural Reform and High-Level Policy Integration

 To institutionalize HL, as a sustainable governance tool, LMIC health system must transcend traditional educational models. Governance structures must be reformed to support advocacy and mediation, thereby addressing the structural determinants of health.

###  1. Institutionalizing Health Literacy Advocacy

 Advocacy serves as the primary driver and engine of systemic reform and a critical mechanism for achieving health equity. It is not merely communication; it is a political and social process designed to influence policy and resource allocation. In centralized health systems common to the MENA region, effective advocacy requires a high-level mandate.

 As articulated in the foundational frameworks of health promotion, advocacy acts as a bridge between individual health needs and structural power dynamics, directly addressing the inequalities that marginalize vulnerable populations.^13^ Effective HL advocacy aligns action across four domains—policy, program, position, and resource allocation. We propose the establishment of a regional Health Literacy Task Force for the MENA region to foster multisectoral coordination and elevate HL from a peripheral activity to a core governance benchmark. Drawing on the Ottawa Charter’s mandates to enable, mediate, and advocate, this task force would oversee program accreditation, enforce intersectoral accountability, and monitor progress at the national level. Such high-level integration ensures that HL initiatives are not siloed within the health system but are embedded across education, media, and social welfare sectors.

###  2. Mediation and the “Health Annex”

 Alongside advocacy, mediation, as one of the three strategic pillars of the Ottawa Charter, ensures that all national policies integrate health and HL considerations. To transition from rhetoric to governance, health systems across the MENA region should institutionalize a “Health Annex” in every major national policy and development plans.

 This mechanism operationalizes the Health in All Policies (HiAP) approach, moving beyond simple intersectoral collaboration to a structured system of accountability. As outlined in the Helsinki Statement, HiAP acts as a framework for country action, requiring that health implications be systematically considered across decisions in sectors other than health.^14^

 To ensure operational feasibility, the “Health Annex” requires a clear institutional mandate, ideally housed within a centralized High Council for Public Health or a Supreme Planning Council with cross-sectoral authority. This body would enforce a mandatory “HL-friendliness” impact assessment prior to policy approval. The enforcement mechanism must be binding: if a development project fails to meet the established health equity and literacy criteria, it must be returned to the proposing ministry for mandatory revision or rejection. This creates a regulatory feedback loop, ensuring that health considerations are not merely advisory but are integral to the legislative process.^15^

 In the LMICs context, the “Health Annex” would serve as a binding regulatory filter. Before approval, development projects would be evaluated for their “HL-friendliness” and impact on community health determinants. This aligns with the theoretical frameworks for HiAP evaluation proposed by Baum et al. (2014), which emphasize that successful governance must not only implement policies but also rigorously assess their implementation logic and impact on health equity.^16^ By mediating these cross-sectoral dynamics, the health system ensures that public policies facilitate, rather than hinder, the population’s ability to make healthy choices.

###  3. Media Advocacy as Governance instrument

 In this governance-oriented HL roadmap in LMICs, media engagement is elevated from a peripheral communication tool to a macro-level policy lever to shape social norms and influence legislative agendas. However, realizing this potential requires acknowledging the dual nature of media in the MENA region, where outlets can serve as accountability tools but may also be subject to state control or censorship.^17^ To navigate this, the roadmap proposes the establishment of specialized “Media Health Advocacy Teams” within the Ministry of Health and medical universities.

 Unlike traditional educational campaigns, these teams function as political and strategic units focused on “Health Advocacy.” Their mandate is to persuade and negotiate with non-health sectors to adopt health-friendly policies. For instance, when intersectoral collaboration stalls, these teams deploy targeted advocacy plans to engage decision-makers and overcome resistance. Unlike traditional educational communicational campaigns, media advocacy operates at the agenda-setting level, as systemic issues that necessitate policy-driven solutions. By positioning media systems as active governance actors rather than mere information channels, the burden of health literacy shifts from individual comprehension to institutional transparency and legislative responsiveness. Operationally, this transformation requires the establishment of structured partnerships between ministries of health and independent media bodies to strengthen governmental accountability. Evidence demonstrates that sustained media framing can accelerate regulatory reforms, influence fiscal prioritization, and mobilize public support for equity-driven policies.^18^ Especially in LMIC contexts—where misinformation often erodes institutional trust—proactive media advocacy serves a stabilizing governance function. By linking policy decisions directly to population health outcomes through public reporting and strategic communication, this framework reinforces the legitimacy of the health system and ensures that institutional accountability remains a permanent feature of the national health roadmap.

## Pillar II: Rebuilding Trust and Mobilizing Social Capital

 Public trust is criteria for effective health governance. Without a foundation of social capital, HL interventions remain theoretical, failing to gain traction in real-world settings. Social capital is significantly associated with a range of positive health outcomes and act as a mediating determinant between governance reforms and population engagement.^19^ A critical distinction between HL frameworks in the high-income countries (HICs) versus the LMICs is the role of trust. In many LMICs, low trust in public institutions is a primary barrier to engagement. Therefore, the PITA framework (Participation, Inclusion, Transparency, and Accountability) is not merely ethical requirements but functional prerequisites for improving service delivery and curbing the corruption that often erodes people’s trust.^20^

###  1. Transparency and Accountability as Governance Mechanisms

 Governance structures must institutionalize transparency mechanisms to reduce information asymmetries that perpetuate mistrust. To ensure inclusivity and mitigate barriers posed by low digital literacy or infrastructure deficits, a multi-channel transparency strategy is essential. This approach should complement high-tech solutions, such as open data portals and national performance dashboards, with low-tech, community-based mechanisms. These include public hearings, community scorecards, and physical information boards displayed at Primary Health Care (PHC) centers, ensuring that transparency is accessible to all citizens regardless of their digital access. Operationally, performance dashboards at national and district levels can display indicators such as patient satisfaction, service accessibility, complaint resolution rates, and HL-responsive service standards. Patient feedback loops —integrated into digital platforms or PHC centers create continuous quality improvement cycles.^21^ Good governance interventions, which include promoting transparency, accountability, and public participation, have shown encouraging results in LMICs.^22^ Thus, transparency is not symbolic; it is a structural instrument for rebuilding institutional legitimacy.

###  2. Institutionalizing Community Participation 

 Leveraging local social fabric is essential for sustainable health outcomes. Successful models, such as the community health worker (CHW) programs seen in Iran, Morocco, and Pakistan, demonstrate the potential of integrating local volunteers and religious networks into the formal health systems. ^23^ However, community participation across the MENA often remains fragmented. Civil society organizations often operate in silos, lacking the coordination required to influence national policy. ^24^ To remedy this, we propose:

- Strategic Networking: Establishing a national database of health Non-Governmental Organizations (NGOs) to facilitate coalition-building.
- Operational Transparency: Mandating financial and operational reporting for NGOs to ensure legitimacy.
- Specialized Training: Providing government-backed training in advocacy and resource management to professionalize the third sector.

Crucially, while professionalization is necessary, it must not come at the cost of civil society autonomy. The relationship between the health system and NGOs must be framed as one of mutual accountability rather than top-down oversight. Safeguards must be established to ensure that government-backed training and reporting mandates are not utilized to co-opt or control independent organizations, thereby preserving their essential role in advocacy and social accountability. By implementing these strategies, the health system can transform NGOs from charitable outliers into essential partners in the national health literacy roadmap.

## Pillar III: Human Resource Empowerment and Systemic Capacity

 A major barrier in transitional health systems is the managerial misconception that HL is solely the patient’s responsibility. This deficit-oriented framing produces adverse consequences: it normalizes communication failure, perpetuates inequitable access, increases medical errors, and contributes to avoidable hospital readmissions. When organizations externalize responsibility to patients, structural barriers remain unaddressed, undermining quality improvement efforts.

###  1. Redefining Professional Competency through Organizational Health Literacy (OHL) 

 We propose the establishment of a regional OHL competency framework, positioning HL as a core institutional responsibility. This framework would redefine quality of care by embedding HL responsiveness into service design, communication standards, and managerial performance evaluation. In this model, health organizations—not patients—are accountable for ensuring comprehensible, navigable, and equitable services.

###  2. Curriculum Reform 

 Transformative HL requires upstream reform of medical and health sciences education. Curricula should be systematically revised to include:

- Health Communication Competency:encouraging for training in Plain Language, shared decision-making, and the Teach-Back Method (verifying understanding by asking patients to repeat information) to institutionalize comprehension verification.
- Systems Thinking: Preparing health professionals to recognize HL as a governance issue and to act as advocates within policy and organizational structures.

Crucially, given the workforce shortages in many LMICs, these competencies must be extended to nurses and community health workers through task-shifting policies. However, for task-shifting to be sustainable and effective, it must be framed within a comprehensive support package for these frontline health professionals. Simply assigning new HL responsibilities without addressing existing structural constraints risks failure. Therefore, policy reforms must be accompanied by measures to manage workloads, ensure fair compensation, and provide clear career progression pathways. By integrating HL training with incentives and professional development, the health system can motivate and empower these essential workers rather than exacerbating their burden.

## Pillar IV: Operationalizing HL in PHC

 The PHC network often the strongest asset in developing health systems and act as an optimal operational hub for HL delivery. PHC platforms are uniquely positioned at the interface between communities and formal health institutions, allowing integration of preventive care, health promotion, and social determinant interventions. The Alma-Ata and Astana Declarations underscore PHC as the foundation of equitable and people-centered health systems. ^25^

###  1. Social Prescribing and Social Determinant of Health (SDH)

 PHC network provides the optimal platform for HL integration. Family physicians should adopt social prescribing, referring patients to community-based resources (e.g., volunteer groups, social support) to address psychosocial determinants. This approach recognizes that medical treatment alone cannot resolve issues rooted in social deprivation or exclusion. Effective social prescribing depends on the presence of a robust ecosystem of community assets—such as NGOs, volunteer groups, and social services—capable of receiving and supporting referrals. Recognizing that such ‘prescribing infrastructure’ is weak or absent in many MENA settings, the roadmap explicitly calls for parallel investments in mapping community resources, sustainable financing mechanisms, and capacity-building programs for community-based organizations to ensure the functionality and equity of social prescribing pathways. This embeds HL in PHC, converting family physicians into connectors between health and society.

###  2. Digital Equity and Reducing the Digital Divide

 Digital transformation must not reproduce inequities. National HL frameworks in the MENA region, must include measures to bridge the digital divide through e-health platforms, telemedicine, and digital education. Efforts must ensure equitable access for marginalized groups, rural populations, and the elderly, recognizing that digital tools can only advance equity if accompanied by infrastructure investment. ^26^

###  3. Performance-Based Payment for HL

 To operationalize HL responsiveness, performance-based payment must rely on specific and measurable indicators, including validated tools for assessing patient comprehension, shared decision-making quality, medication literacy, and adherence to self-management plans. Payments should be structured using risk-adjustment mechanisms—such as socioeconomic and clinical complexity indices—to prevent penalizing PHC teams serving disadvantaged or hard-to-reach populations. The framework also incorporates safeguards against unintended consequences, including data manipulation, patient cherry-picking, and over-standardization, through independent audits, equity-weighted scoring, and blended payment models.

## Pillar V: Sustainable Financing

 The final pillar for Building health literacy system capacity, positioning health literacy financing as a core component of fiscal governance rather than as isolated program funding. Health literacy investments and financial stewardship are recognized as one of eight action areas for structural transformation. ^27^ Without institutionalized fiscal governance mechanisms, governance reforms remain aspirational. We propose earmarking sin taxes on unhealthy products (tobacco, sugary drinks) to finance HL and health promotion projects. This approach advocated by the WHO Fiscal Policies for Health, embeds HL within national budgetary and revenue-allocation processes, creating a virtuous cycle in which revenue generated from health-harming products is systematically reinvested in preventive capacity and population empowerment. However, the implementation of such fiscal policies is rarely straightforward; it is often contested by powerful industries (e.g., tobacco and sugar lobbies) that fiercely oppose taxation. To overcome this political economy challenge, the proposed HL Task Force and Health Advocacy Teams must build strong cross-sectoral coalitions to generate the necessary political will and public support.

 To protect the integrity of HL financing, safeguards against fungibility are necessary. Earmarked revenues must be structured to supplement rather than substitute existing health allocations through legally binding budget rules, dedicated HL and health-promotion funds, and independent monitoring of revenue flows. These measures ensure that fiscal policies translate into real, additional investments in preventive capacity and population empowerment, rather than reallocation of existing spending.

## Conclusion and Policy Implications

 The institutionalization HL, as a governance mechanism represents a fundamental paradigm shift, moving beyond the traditional view of HL as an individual deficit to recognizing it as a structural determinant of health equity and a core component of responsive health systems. For the MENA region and similar LMICs, reliance on imported frameworks is insufficient; instead, the region requires a context-grounded architecture that addresses specific challenges such as centralized authority, fragmented coordination, and constrained fiscal space. The proposed five-pillar roadmap—encompassing high-level policy advocacy, the rebuilding of social capital, workforce competence, primary care integration, and sustainable financing—provides a cohesive strategy to embed HL into the fabric of the health sector. By operationalizing these pillars, HL is transformed from a peripheral educational activity into a binding logic of governance that ensures accountability and operational transparency.

 Ultimately, the strength of this framework lies in its systemic coherence, creating a virtuous cycle wherein policy integration fosters public trust, which in turn drives community engagement and justifies sustained investment. This model serves not merely as a rigid prescription, but as a transferable template adaptable to the diverse political and cultural specificities of the MENA region. Realizing a health-literate society is fundamentally an exercise in political will, acknowledging that health systems function as shapers of citizenship. The central question is no longer whether MENA health systems can afford to implement this governance roadmap, but whether they can afford the continued political and social cost of not doing so—a cost measured in eroded trust, perpetuated inequity, and preventable morbidity. The choice, as the Shanghai Declaration asserts, is fundamentally a political one.

## Competing Interests

 The Authors declares that there is no conflict of interest.
